# *Schistosoma haematobium* hotspots in south Nyanza, western Kenya: prevalence, distribution and co-endemicity with *Schistosoma mansoni* and soil-transmitted helminths

**DOI:** 10.1186/1756-3305-7-125

**Published:** 2014-03-25

**Authors:** Huldah C Sang, Geoffrey Muchiri, Maurice Ombok, Maurice R Odiere, Pauline NM Mwinzi

**Affiliations:** 1 Center for Global Health Research, Kenya Medical Research Institute, P.O. Box 1578-40100, Kisumu, Kenya

**Keywords:** Schistosomiasis, Soil transmitted helminths, Geographical distribution, South Nyanza, Kenya

## Abstract

**Background:**

Schistosomiasis studies in western Kenya have mainly focused on the intestinal form, with evidence of urinary schistosomiasis remaining anecdotal. Detailed disease mapping has been carried out predominantly along the shores of Lake Victoria, but there is a paucity of information on intestinal and urinary schistosomiasis in inland sites.

**Methods:**

This cross-sectional survey of 3,487 children aged 7–18 years from 95 schools in south Nyanza, western Kenya determined the prevalence, infection intensity, and geographical distribution of *Schistosoma haematobium,* evaluating its co-endemicity with *Schistosoma mansoni* and soil-transmitted helminths (STHs). Helminth eggs were analyzed from single urine (for *S. haematobium*) and stool (for *S. mansoni* and STHs) samples by centrifugation and Kato-Katz, respectively. Hematuria was used as a proxy indicator for *S. haematobium*. Schools and water bodies (ponds, water-points, streams, dams and rivers) were mapped using Geographical Information System and prevalence maps obtained using ArcView GIS Software.

**Results:**

*S. haematobium* infections with an overall prevalence of 9.3% (95% CI = 8.4-10.2%) were mostly prevalent in Rachuonyo, 22.4% (95% CI = 19.2-25.9% and 19.7 eggs/10 ml) and Migori, 10.7% (95% CI = 9.2-12.3% and 29.5 eggs/10 ml) districts, particularly around Kayuka pond and Ongoche river respectively. Overall infections correlated with hematuria (r = 0.9, P < 0.0001) and were more likely in boys (P < 0.0001, OR = 0.624). *S. mansoni* infections with an overall prevalence of 13% (95% CI =11.9-14.1%) were majorly confined along the shores of Lake Victoria. STH infections were homogenously distributed with *A. lumbricoides* occurring in 5.4% (95% CI = 4.7-6.3%) and *T. trichiura* in 2.8% (95% CI = 2.3-3.4%) of the children. Although *S. mansoni* infections were more co-endemic with *S. haematobium*, only *A. lumbricoides* infections were positively associated with *S. haematobium* (P = 0.0295, OR = 0.4585). Overall prevalence of *S. haematobium* monoinfection was 7.2% (95% CI = 6.4-8%), *S. mansoni* monoinfection was 12.3% (95% CI = 10.4-12.5%), and *S. haematobium*-*S. mansoni* coinfection was 1.2% (95% CI = 0.9-1.6%). There was no significant difference in infection intensity between mono and coinfections.

**Conclusion:**

Prevalence distribution maps obtained are important for planning and implementing disease control programs in these areas.

## Background

Schistosomiasis is a major yet neglected public health problem caused by trematodes of the genus *Schistosoma*[1]. An estimated 243 million people are affected globally, with 85% of these cases living in Sub-Saharan Africa, including Kenya, where nearly 6 million people are infected and an additional 15 million are at high risk of infection particularly in endemic areas [2-5]. The two main species of concern in Kenya are *Schistosoma haematobium,* the cause of urinary schistosomiasis and *Schistosoma mansoni,* the cause of intestinal schistosomiasis [2]. Chronic infection causes adverse morbidity-related effects that are exacerbated by multiple species infections and high parasite loads [6].

Control programmes aimed at reducing morbidity employ mass drug administration (MDA) strategies in treating infected populations with the drug Praziquantel [7,8]. Such interventions rely on precise geographical identification of parasite transmission areas through quantification of disease prevalence and infection intensities amongst the at-risk populations. Geographic distribution of disease largely depends on the climatic and environmental factors essential for the presence of the *Biomphalaria* spp. and *Bulinus* spp. snails, intermediate hosts for *S. mansoni and S. haematobium* respectively [9]. Earlier studies carried out in the south Kenyan Coast and western Kenya show that together with other prevailing factors, terrestrial aquatic environments such as ponds, streams, swamps, rivers and to a less extent dams are the main inland *S. haematobium* transmission sites [9-11]. Nonetheless, human infection pattern varies depending on how pre-disposed individuals come into contact with snail-infested water in their daily socio-economic activities [12].

Global schistosomiasis distribution maps show a large overlap of both *S. mansoni* and *S. haematobium* infections in endemic areas, portraying the risk of co-infection amongst the pre-disposed population [2,13,14]. Extensive studies done in Nyanza province, western Kenya, strongly point to a high prevalence of *S. mansoni* particularly confined along the shores of Lake Victoria, with prevalence decreasing further away from the lake [15,16]. Although this has been established, the distribution of *S. haematobium* and its potential transmission hotspots has not yet been fully elucidated. Earlier studies carried out in Asembo area (northern part of the lake shore) and within the informal settlements of Kisumu city found hardly any cases of *S. haematobium* infection [15,17]. However, symptoms of urogenital schistosomiasis such as passing bloody urine (hematuria) have been observed in school going children and even young adults in some areas close to inland water bodies (ponds, water-points, streams, dams or rivers) within south Nyanza region, suggesting potentially high *S. haematobium* endemicity. This situation is further exacerbated by the low socioeconomic and hygiene standards including inadequacy of potable water supply, lack of proper medical attention, poor sanitation, ignorance and poverty, collectively known to have direct impact on disease distribution [18].

In line with the emphasis laid by the World Health Organization (WHO) in creating predictive *Schistosome-*distribution maps, Geographic Information Systems (GIS) is employed in mapping the spatial patterns of human infection. This is vital for the proper planning, surveillance and implementation of effective MDA interventions [19-22]. In this regard, the aim of this cross-sectional study was to determine *S. haematobium* prevalence, infection intensity, geographical distribution and its co-endemicity with *S. mansoni* and other soil-transmitted helminths (STHs) amongst primary school going children in south Nyanza (Homabay, Migori, part of Nyamira and Kisii counties). A proper understanding of disease prevalence and infection intensities will not only provide a useful tool for proper planning of effective control programmes but also form a basis of exploring other potential adverse health related effects instigated by *S. haematobium,* including female genital schistosomiasis (FGS) and cervical carcinoma*.*

## Methods

### Study area

This was a cross-sectional study conducted in south Nyanza, western Kenya between May - June 2013. This region currently falls under four counties i.e. Homabay, Migori, Kisii and Nyamira with the co-ordinates 0° 31'S and 34° 27'E, 1° 3'S and 34° 28'E, 0° 40'S and 34° 46'E , 0° 38'S and 34° 58'E respectively. Counties are further divided into districts. This study was carried out in 7 districts namely Borabu, Central Kisii, Gucha South, Homabay, Migori, Rachuonyo and Suba.

The rainfall pattern in this region is generally bimodal. Homabay and Migori counties (bordering L. Victoria to the north and west) experience less annual mean rainfall (250 mm - 700 mm) with higher mean temperatures (17°C - 35°C) compared to Kisii and Nyamira counties (600 mm – 2300 mm, 10.1°C - 28.7°C) which are located on the highlands. This kind of climate favours subsistence farming which is the main economic activity alongside fishing. Besides its economic importance, L. Victoria is the main water source for nearly all the domestic and socioeconomic activities of the nearby inhabitants. Those living further away depend on water from open boreholes, ponds, streams, dams or rivers.

### Study design

Ninety five public primary schools were selected for enrollment based on close proximity (within a radius of 4 km) to permanent/semi-permanent ponds, dams, water-points, rivers or lake obtained from Google maps. Fifty pupils were randomly selected via a random number generator in each school. Informed consent and assent to participate in the study was sought from the parent and pupil respectively. Ethical clearance for the study was obtained from the Ethical and Scientific Steering Committee of the Kenya Medical Research Institute (KEMRI). The Institutional Review Board of the Centers for Disease Control and Prevention also reviewed the study and chose to rely on the KEMRI ERC approval. Prior to this study, a school based national deworming with a single dose of albendazole (400 mg) had been conducted in September 2012, but there had not been any mass drug treatment with praziquantel.

### Parasitological assessment

A single urine and stool sample from each child was collected between 10.00 am and 2.00 pm. Samples were stored cool in dark plastic bags and transported to the laboratory for processing the same day. Hematuria in urine was determined using urine reagent strips (YD Diagnostics Corporation) and the results scored within 60 seconds. Ten milliliter homogenized urine samples were centrifuged (BECKMAN model TJ-6 centrifuge) at 1,714 g for 3 minutes and the supernatant drawn off. *S. haematobium* infection was determined by egg count microscopy and categorized as per the WHO quantification guidelines as mild (1–49 egg/10 ml urine) or heavy (≥50 eggs/10 ml urine) [23]. Individual stool samples were processed in duplicate according to the Kato-Katz technique on the same day, slides were left to clear for at least 24 hours, kept in slide boxes at room temperature and then microscopically examined for the presence of *S. mansoni, Trichuris trichiura* and *Ascaris lumbricoides* ova within a month. *S. mansoni* infection intensity was classified as either mild (< 100 epg), moderate (100–399 epg), or heavy (≥400 epg) while STH infections were scored as either positive or negative [23].

The following definitions are used in this paper: overall parasite infection denotes infection with a single parasite species with or without other infections, monoinfection denotes infection with only a single species and coinfection denotes infection with both *S. haematobium* and *S. mansoni.*

### Geographical distribution

In order to establish the geographical location of infection according to its prevalence, the coordinates of inland water bodies (ponds, water-points, streams, dams or rivers) and selected schools were obtained using a hand-held global positioning system device (GPS) unit (Trimble navigation Ltd, California USA). Coordinates of schools or water-bodies that were incomplete or could not be obtained from site visits were manually searched using the European Union GPS coordinates finder (http://www.gpscoordinates.eu/). Data sets on prevalence were imported to a GPS database (GPS pathfinder office 2.8 Trimble Navigation Ltd, California, USA) and plotted using the GIS software ArcView version 9.2 (Environmental Systems Research Institute, Inc., Redlands, CA). For schools located within a 25 km distance from the lake, the shortest distance from a school to the lake was calculated using ArcMap version 9.2. Mapped prevalence was categorized according to WHO infection prevalence thresholds for MDA; 0.1-9.9%, 10–49.9% and 50-100%. An additional one, 0%, was added to our maps [23].

### Data analysis

Data on parasitological assessment was analyzed using Graph Pad Prism version 5 and all results with a P value of < 0.05 were considered significant. Intensity of infection for both *S. haematobium* and *S. mansoni* was calculated based on arithmetic mean egg counts. Spearman correlation was used to determine the correlation between schistome spp. infection and distance from the lake as well as *S. haematobium* infection and hematuria. The Mann–Whitney test was used to compare the difference in infection intensities between girls and boys. Logistic regression was used to assess the relation between hematuria or *S. haematobium* infection and gender. Chi square test was used to assess the difference between *S. haematobium* and *S. mansoni* prevalence and to determine the association between *S. haematobium* infections with the other parasite species infections. The Mann–Whitney test was used to compare *S. haematobium and S. mansoni* mono versus coinfection intensities.

## Results

### Geographical distribution of infections

A total of 3,846 children from 95 schools who submitted their urine samples were recruited in the study with a mean age of 13.4 and a median of 13 (range 7 – 18) years. Only 3,420 (88.9%) pupils returned their stool sample.

**Figure 1:**
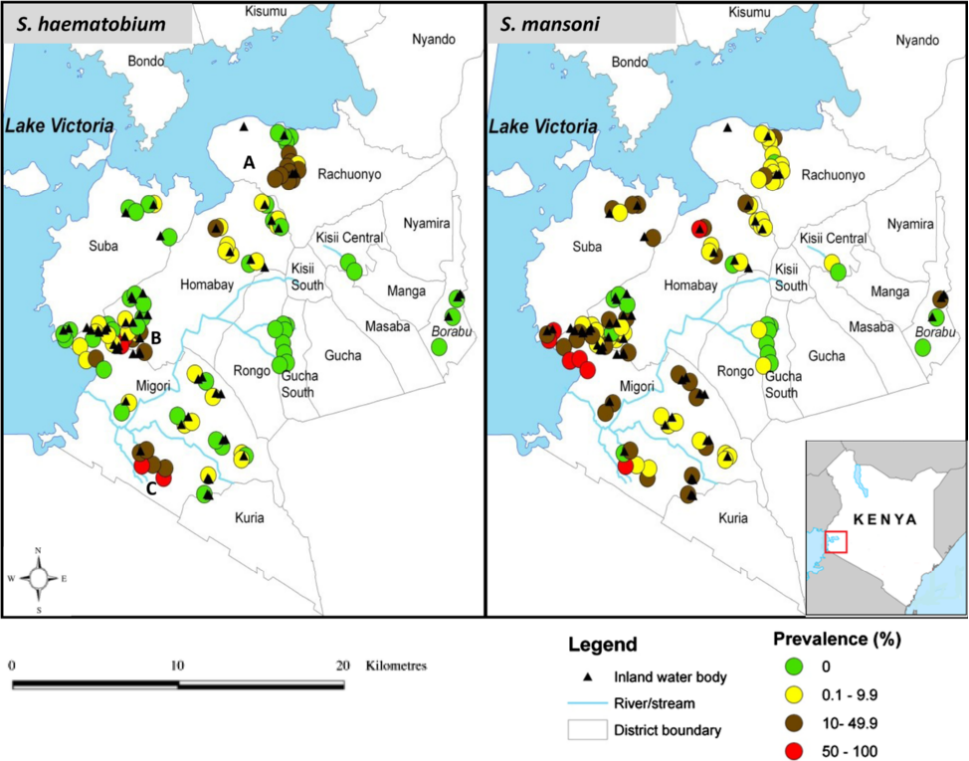
**Prevalence of *Schistosoma haematobium *** **and *Schistosoma mansoni *** **in selected primary schools in south Nyanza, western Kenya.**

**Figure 2:**
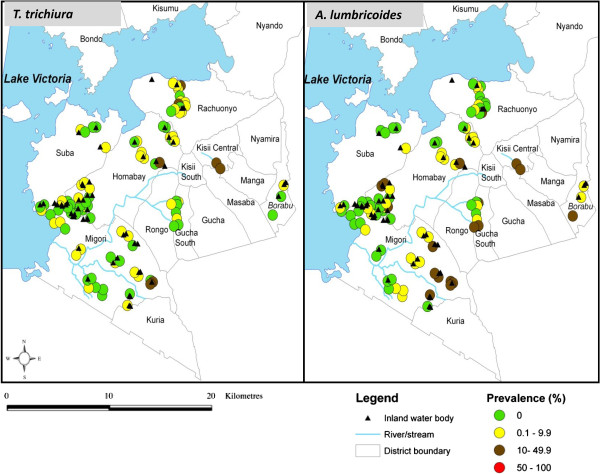
**Prevalence of *Trichuris trichiura *** **and *Ascaris lumbricoides *** **in selected primary schools in south Nyanza, western Kenya.**

*S. haematobium* infections, mainly occurring near inland water bodies (ponds, water-points, streams, dams or rivers), were found in a smaller number of schools (49 schools) compared to *S. mansoni* infections (78 schools), that were majorly confined along the shores of the lake (Figure 1). Distance from the lake was inversely associated with *S. mansoni* prevalence (*r* = −0.7, P = 0.0004), but was positively associated with *S. haematobium* prevalence (*r* = 0. 5, P = 0.008). STH infections were more homogeneously distributed with *A. lumbricoides* occurring in 51 schools and *T. trichiura* in 49 (Figure 2).

### Overall prevalence

The overall prevalence of hematuria and *S. haematobium* were 8.3% and 9.3%, respectively. *S. mansoni* infections were found in 13% of the children (Table 1). Prevalence of ≥ 10% for both *S. haematobium* and *S. mansoni* infections were found in 22 schools (23.2%) and 37 schools (38.9%) respectively, with 7 (7.4%) and 9 schools (9.5%) recording prevalence of ≥ 50%. STH prevalence was ≥ 10% in 26 schools (27.4%), out of which 9 schools (9.5%) had ≥ 20% prevalence. No school had ≥ 50% STH infection. The prevalence of hematuria and parasite infection in all the schools stratified to their respective districts was further assessed.

**Table 1:** Prevalence and intensity of infection of schistosomiasis and soil-transmitted helminths among school children in south Nyanza, western Kenya

	**Overall prevalence (%)**^**1**^	**Intensity threshold prevalence, (%)**			***Infection intensity *** **(eggs/10 ml or epg)**^**2**^
		**Mild**	**Moderate**	**Heavy**	
**Hematuria**	8.3 (7.5-9.2)				
**Overall parasite infection**					
*S. haematobium*	9.3 (8.4-10.2)	86.2	-	13.8	24.1 (18.7-29.6)
*S. mansoni*	13 (11.9-14.1	63.2	24.2	12.6	156.8 (135.3-178.4)
*T. trichiura*	2.8 (2.3-3.4)				
*A lumbricoides*	5.4 (4.7-6.3)				
**Monoinfection**					
*S. haematobium*	7.2 (6.4-8)	86.6	-	13.4	24 (17.2-30.7)
*S. mansoni*	11.4 (10.4-12.5)	62.3	25.1	12.6	159.2 (136.7-181.7)
**Coinfection**					
*S. haematobium* - *S. mansoni*	1.3 (0.9-1.6)	78^3^	29.3^4^	22^3^	26.6 (16.2-37.0)^3^
		68.3^4^		2.4^4^	92.8 (63.3-122.3)^4^

^1^95% Confidence interval.

^2^Arithmetic mean infection intensity (values in parenthesis indicate 95% Confidence Interval).

^3^Values corresponding to *S. haematobium* infections.

^4^Values corresponding to *S. mansoni* infections.

### Prevalence of hematuria and *S. haematobium* infections

Hematuria was most prevalent among children in Rachuonyo (14%) and Migori districts (9.5%) (Table 2). School prevalence for hematuria strongly correlated with those of *S. haematobium* infections (*r* = 0.9, P < 0.0001). Infection mainly occurred in three districts: Rachuonyo (22.4% and 19.7 eggs/10 ml), Migori (9.5% and 29.7 eggs/10 ml) and Homabay (6.2% and 18.6 eggs/10 ml). Importantly, higher school prevalence was noted in 3 areas; around Kayuka pond and Kamenya dam (A in Figure 1) in Rachuonyo district (prevalence range = 20% - 44%), a water point (B) in Homabay district (prevalence range = 23% - 48%) and Serikali pond (part of B) and along the Ongoche river (C) in Migori district (prevalence range = 23% - 80%). This is suggestive of most likely *S. haematobium* transmission hotspots.

**Table 2:** **Prevalence and intensity of infection of hematuria and *Schistosoma haematobium *** **infections among school children by District in south Nyanza, western Kenya**

**District**	**No of schools**	**No of pupils**	**Hematuria prevalence (%)**^**1**^	***S. haematobium *** **prevalence (%)**^**1**^	**Intensity threshold prevalence, (%)**		**Mean intensity (eggs/10 ml)**^**2**^
					**Mild**	**Heavy**	
**Borabu**	3	118	0.8 (<0.01-5.1)	0	0	0	0
**Central Kisii**	2	66	3 (0.2-11)	0	0	0	0
**Gucha South**	8	339	5 (3.1-7.9)	0	0	0	0
**Homabay**	26	1029	6.2 (4.9-7.9)	5.7 (4.5-7.3)	88.1	11.9	18.6 (12.7-24.5)
**Migori**	37	1546	9.5 (8.1-11.1)	10.7 (9.2-12.3)	83.0	17	29.7 (19-40.5)
**Rachuonyo**	14	586	14 (11.4-17.1)	22.4 (19.2-25.9)	89.3	10.7	19.7 (14.4-25)
**Suba**	5	162	4.3 (2-8.8)	0.6 (<0.01-3.8)	100	0	12
**Overall**	95	3846	8.3 (7.5-9.2)	9.3 (8.4-10.2)	86.2	13.8	24.1 (18.7-29.6)

^1^95% Confidence Interval.

^2^Arithmetic mean infection intensity (values in parenthesis indicate 95% Confidence Interval).

*S. haematobium* infections were dominantly mild, with up to 17% heavy infections occurring in Migori. The difference in mean infection intensities between girls and boys was insignificant. Pupils with hematuria were more likely to have *S. haematobium* infection (P < 0.0001). Notably, not all the *S. haematobium*-infected participants had hematuria and vice versa. Out of the 320 hematuria positive children, 60% (190/320) tested positive for *S. haematobium,* whereas 47% (166/356) of the *S. haematobium* positive participants tested negative for hematuria. Therefore, only 4.9% (190/3846) of the overall *S. haematobium* infected children had hematuria.

Moreover, analysis based on gender revealed that although hematuria was recorded in slightly higher numbers of girls 52.8% (169/320) than boys 47.2% (151/320), it was not significantly associated with gender. Nonetheless, *S. haematobium* infection was more likely in boys than girls (P < 0.0001, OR = 0.624).

### Prevalence of *S. mansoni* and STHs

Prevalence of *S. mansoni* and STH infections for all the districts showed that infections were mostly prevalent amongst children in Migori (21.4% for *S. mansoni*) and Central Kisii district (12% for *T. trichiura* and 23.8% for *A. lumbricoides*) (Table 3). Notably, *S. mansoni* infections were significantly more prevalent than *S. haematobium* overall (P = 0.0001) except in Rachuonyo district, where the prevalence of *S. haematobium* (22.4%) was nearly four times that of *S. mansoni* (6.1%) (Table 3).

**Table 3:** **Prevalence and intensity of infection of *Schistosoma mansoni *** **and soil-transmitted helminths among school children by is District in south Nyanza, western Kenya**

**District**	***S. mansoni *** **prevalence (%)**^**1**^	**Intensity threshold prevalence, (%)**			**Mean intensity (epg)**^**2**^	***T. trichiura *** **prevalence (%)**^**1**^	***A. lumbricoides *** **prevalence (%)**^**1**^
		**Mild**	**Moderate**	**Severe**			
**Borabu**	3.7 (1.1-9.4)	100	0	0	21 (2.7-39.2)	0.9 (<0.01-5.6)	6.5 (3–13)
**Central Kisii**	2.4 (<0.01-13.4)	100	0	0	36	12 (4.7-25.5)	23.8 (13.3-38.7)
**Gucha South**	0.3 (<0.01-2.1)	100	0	0	24	1.7 (0.6-4)	11.7 (8.5-15.8)
**Homabay**	8.6 (6.9-10.6)	73.4	20.3	6.3	118.1 (69.7-166.5)	2.6 (1.7-3.9)	4.9 (3.7-6.5)
**Migori**	21.4 (19.3-23.6)	58.8	25.6	15.6	179.4 (151.4-207.4)	2.6 (1.9-3.5)	5.4 (4.3-6.7)
**Rachuonyo**	6.1 (4.3-8.5)	67.7	22.6	9.7	115.5 (58.6-172.4)	4.1 (2.7-6.2)	2.3 (1.3 to 4.1)
**Suba**	19.4 (13.6-27)	69.2	26.9	3.8	93.2 (44.9-141.6)	2.3 (0.5-6.7)	0.7 (<0.01-4.5)
**Overall**	13 (11.9-14.1	63.2	24.2	12.6	156.8 (135.3-178.4)	2.8 (2.3-3.4)	5.4 (4.7-6.3)

^1^95% Confidence Interval.

^2^Arithmetic mean infection intensity (values in parenthesis indicate 95% Confidence Interval).

*S. mansoni* infections were also dominantly mild and moderate, with about 16% heavy infections occurring in Migori district. The difference in mean infection intensities between girls and boys was not significant. There was no association between *S. mansoni* infection and gender.

### Co-endemicity of *S. haematobium* with *S. mansoni* and STHs

Out of the 1, 080 infected children, single infection with either of the four parasite species occurred in 990 (91.7%) children, dual and triple species infection was recorded in 85 (7.9%) and 5 (0.5%) children respectively. None of the children were infected with all four helminth species. Of those with single helminth infections, the most dominant infection was *S. mansoni* (45.7%), followed by *S. haematobium* (30.1%), *A. lumbricoides* (17.6%) and lastly *T. trichiura* (6.7%). Amongst the dual species infections, *S. haematobium* coinfection with other parasites occurred in 56 (16.9%) out of the 331 *S. haematobium* positive children. Analysis based on prevalence of other parasitic infections in children with or without *S. haematobium* infections showed that *A. lumbricoides* infections were significantly associated with *S. haematobium* (P = 0. 0295) (Table 4)*.* Dual *S. mansoni* coinfections occurred in 84 (19.1%) out of the 438 *S. mansoni* positive children. Coinfection with *S. haematobium* dominated by 9.4% (41/438), then *T. trichiura* at 3.9% (17/438) and *A. lumbricoides* at 2.3% (10/438). Only *A. lumbricoides* infections were associated with *S. mansoni* infections (P = 0. 0026). Importantly, there was no association between infection with *S. mansoni* and *S. haematobium* and vice versa.

**Table 4:** **Prevalence of other parasitic infections in children with or without *S. haematobium *** **infection in south Nyanza, western Kenya**

	***S. haematobium *** **positive N = 331**		***S. haematobium *** **negative N = 3063**		**P value**
	**n**	**%**	**n**	**%**	
***S. mansoni***	41	12.4	397	13	0.8338
***T. trichiura***	6	1.8	89	2.9	0.3321
***A. lumbricoides***	9	2.7	176	5.7	0.0295

**Figure 3:**
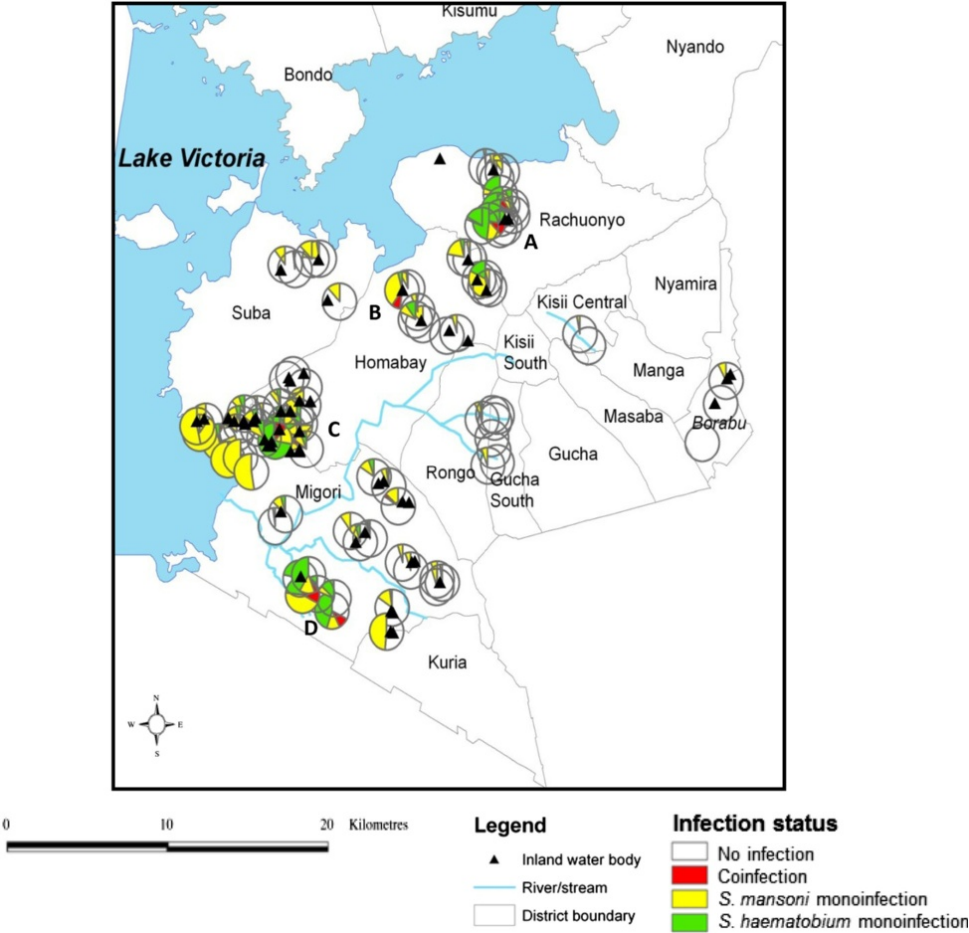
**Prevalence of *S. haematobium *** **and *S. mansoni *** **mono and coinfection in selected primary schools in south Nyanza, western Kenya.**

Spatial heterogeneity of *S. haematobium - S. mansoni* mono versus coinfections revealed that coinfections occurred near Kayuka pond and Kamenya dam (A in Figure 3) in Rachuonyo district, Katumo pond (B), Osani and Wachara pond (C) in Homabay district, and along the Ongoche River (D) in Migori district (Figure 3). *S. mansoni* monoinfections, just like *S. mansoni* overall infections, dominated in all districts compared to *S. haematobium* monoinfections except in Rachuonyo, where the prevalence of *S. haematobium* (18.6%) was nearly four times that of *S. mansoni* (4.9%) (Table 5)*.* Notably, *S. haematobium - S. mansoni* coinfections were uncommon (1.3%) amongst the *S. haematobium* (7.2%) and *S. mansoni* (11.4%) monoinfected children. Assessment of infection intensities revealed that *S. haematobium* intensity was slightly higher in coinfections than in monoinfections, whereas *S. mansoni* intensity was higher in monoinfections than coinfections, though the difference was not statistically significant in both cases.

**Table 5:** **Prevalence and intensity of infection of *Schistosoma haematobium *** **and *Schistosoma mansoni *** **mono and coinfections among school children by District in south Nyanza, western Kenya**

**District**	***S. haematobium *** **monoinfection**		***S. mansoni *** **monoinfection**		**Co-infection**		
	**Prevalence (%)**^**1**^	**Mean intensity (eggs/10 ml)**^**2**^	**Prevalence (%)**^**1**^	**Mean intensity (epg)**^**2**^	**Prevalence (%)**^**1**^	***S. haematobium *** **intensity (eggs/10 ml)**^**2**^	***S. mansoni *** **intensity (epg)**^**2**^
**Borabu**	-	-	2.8 (0.6-8.2)	16 (−1.2-33.2)			
**Central Kisii**	-	-	2.4 (<0.01-13.4)	36	-	-	-
**Gucha South**	-	-	0.3 (<0.01-2.1)	24	-	-	-
**Homabay**	4.5 (3.4-5.9)	17.9 (10.6-25.1)	8.2 (6.6-10.1)	112.2 (62.5-161.9)	0.3 (0.1-0.9)	21.5 (−13.6-56.6)	81 (−75.2-237.2)
**Migori**	7.8 (6.5-9.2)	31.5 (17.2-45.9)	20.5 (18.5-22.7)	183.8 (154.8-212.8)	2.1 (1.4-3)	29.9 (16.1-43.6)	81.9 (53.7-110)
**Rachuonyo**	18.6 (15.7-22)	18.3 (12.7-23.9)	4.9 (3.3-7.2)	113 (48.8-177.1)	8.9 (4.6-16.3)	18.7 (−3.5-40.8)	132 (23.3-240.8)
**Suba**	0.6 (<0.01-3.8)	12	19.4 (13.6-27)	93.2 (44.9-141.6)	-	-	-
**Overall**	7.2 (6.4-8)	24 (17.2-30.7)	11.4 (10.4-12.5)	159.2 (136.7-181.7)	1.3 (0.9-1.6)	26.6 (16.2-37.0)	92.8 (63.3-122.3)

^1^95% Confidence Interval.

^2^Arithmetic mean infection intensity (values in parenthesis indicate 95% Confidence Interval).

**Figure 4:**
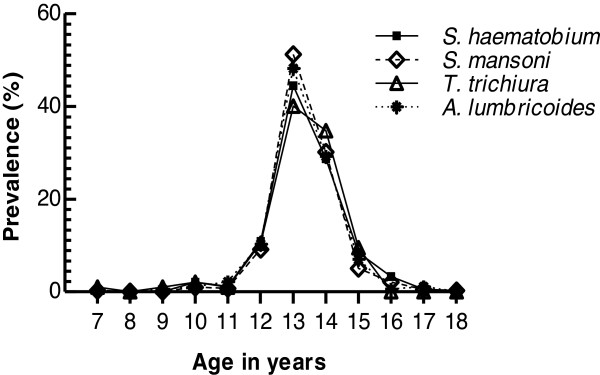
**Prevalence of *S. haematobium*, *S. mansoni *** **and STHs in south Nyanza, western Kenya by age.**

Analysis of infection prevalence of all the helminth species assessed based on the children’s age depicted a gradual increase of infection prevalence from the age of 7–10 years which peaked at 11–14 years then gradually declined at 15–18 years (Figure 4).

## Discussion

Results obtained in this study augments the schistosomiasis and STH baseline survey recently carried out for national deworming in Kenya by Mwandawiro and others [24], covering four districts in south Nyanza. We showed the prevalence distribution of *S. haematobium, S. mansoni* and STH infections in seven districts, pointing out the *S. haematobium* hotspots.

*S. haematobium* transmission hotspots were mainly in Rachuonyo and Migori districts, near the Kayuka pond and Ongoche River respectively. Infection with *S. haematobium* was associated with *A. lumbricoides* and not *S. mansoni* nor *T. trichiura*, despite the more prevalent *S. mansoni* infections.

### Prevalence of hematuria and *S. haematobium*

Chronic *S. haematobium* infection is manifested clinically as anemia, hematuria, dysuria, and urinary tract pathological lesions. This is as a result of schistosome eggs traversing the epithelial walls of the bladder, and is exacerbated by longer exposure periods [25-27]. Egg counts in urine and hematuria are indirect measures of assessing infection and pathology respectively [7]. The results of the present study indicated an overall hematuria prevalence of 8.3%, similar to a prevalence of 7.5% obtained in a study involving 4,901 children in southern Sudan [28]. Prevalence of hematuria varied according to infection status; with a strong association between hematuria and *S. haematobium* infection, consistent with previous studies from the south Kenyan Coast and South Africa [29,30]. Taking gender into account, our findings showed a higher proportion of girls (53%) tested positive for hematuria than boys (47%), agreeing with other studies [31], despite the higher number of boys compared to girls recruited into the study (1,955 boys and 1,891 girls).

The observed lower percentage of *S. haematobium* infected children (4.9%) out of those testing positive for hematuria (8.3%) in our findings is in line with reports from Nigeria where out of the 6% hematuria positive children, only 3.6% were infected [31]. Such discrepancies can be explained from two dimensions. First, there can be false positives especially in girls due to menstruation, as previously reported in Sudan [28]. Second, there are other physiological causes of hematuria besides the pathological lesions instigated by *S. haematobium* infection [32]. Importantly, the observed 47% (166/356) infected children who tested negative for hematuria in our study possibly suggests infection could be at its early stages where chronic clinical symptoms are not yet manifested.

Our overall *S. haematobium* prevalence (9.3%) differed with studies from the south Kenyan Coast showing higher prevalences of 53.8%, [33], 32.4% [34], 55.7%, 43.2% [35] 14% [29] and recently 14.8% [24]. This can be attributed to the focal nature of schistosomiasis distribution [36-38], a vast survey area including 7 administrative districts in our study versus particular *S. haematobium* transmission sites within three villages in the Coastal studies. Such low prevalences, however, were in line with those of large scale screening studies from Sierra Leone (median prevalence of 2% and infection intensity of 3.98 eggs/10 ml urine) and Ghana (7.8% for males, 6.6% for females) [36,39].

However, particular survey sites in the present study had prevalences of ≥ 40%, (with infection intensities of ≥ 20 eggs/10 ml urine) i.e. around Kayuka pond and Kamenya dam in Rachuonyo district, a water point (near Nyandemra primary, Homabay district), a pond (near Ungoe primary, Migori district) and River Ongoche (near Obembo primary, Migori district). Similar to the other schools we surveyed, these high - prevalence schools were within a 4 km radius from a water body (pond, water-point, stream, dam or river), a risk factor for *S. haematobium* transmission [34,40,41]. Combinations of other factors possibly play a role in promoting high prevalence of *S. haematobium* in these particular sites compared to the others in this study. First, visits to individual schools revealed more marshy immediate surroundings and lime type soils that form shallow water lodges when people/cattle pass, especially during rainy seasons. These could act as additional reservoirs for the *Bulinus* spp. snails [10,42]. Second, it is most likely that variation in environmental conditions necessary for breeding of snails could affect snail distribution in our study, considering the fact that in western Kenya, *Bulinus* spp. snails are known to be associated with any form of vegetation excluding the hippo type of vegetation in terrestrial aquatic habitats [10]. Third, Ofulla and others [10] showed a significant difference in the abundance of both *Bulinus* spp. and *Biomphalaria* spp. snails in different locations on land. Furthermore, a recent study has shown significant genetic differentiation in *Bulinus globosus* snails around the L. Victoria basin, suggestively shaped by the seasonality of water bodies [42]. Further investigations could explore more on the malacological status of the transmission sites identified in the present study.

### Prevalence of *S. mansoni* and STHs

Our overall prevalence for *S. mansoni* (nearly 13%) was lower than that observed within the informal settlements of Kisumu city (21%), possibly due to the focal nature of schistosome distribution. In line with other studies, *S. mansoni* infections were majorly more prevalent in schools close to the lake whereas *S. haematobium* infections, concentrated in schools near inland water bodies (ponds, water-points, streams, dams or rivers), [11,16,43,44]. Higher prevalence of *S. mansoni* compared to *S. haematobium* in all the districts except in Rachuonyo can be attributed to the reported significantly higher number of *Biomphalaria spp*. snails than *Bulinus spp*. snails in different terrestrial aquatic habitats in western Kenya [10,11]. The homogenous distribution of STHs in our study (overall prevalence of 5.4% for *A. lumbricoides* and 2.8% for *T. trichiura*) is in tandem with previous studies from western Kenya, although slightly higher percentage prevalence was noted within the informal settlements of Kisumu (4.9% for *A. lumbricoides* and 7.7% for *T. trichiura*) [17]. In line with a recent STH survey in these regions, *A. lumbricoides* infections were more prevalent compared to *T. trichiura*[24].

The observed variation in infection prevalence by district can be attributed to the geographical differences that favour stagnant water conditions. Central Kisii, Gucha South and Borabu districts are generally hilly with higher annual amounts of rainfall, a risk factor associated with lower *S. mansoni* prevalence [45]. On the other hand, Migori, Rachuonyo, Homabay and Suba districts are more or less flat, with more , swamps, streams or dams which mostly dry up during dry seasons due to lower annual amounts of overall rainfall, favoring breeding of snails [10].

Considering age as an infection risk factor, our findings were in agreement with previous studies showing high infection prevalence in 11–14 year old school going children, more so in boys compared to girls, due to their more adventurous habits i.e. drinking contaminated water while grazing cattle, swimming, fishing, playing, bathing, hunting for snails and other activities [46-48]. This finding, however, differed with other studies that reported similar prevalence between both sexes [24,49,50] and higher prevalence in girls than boys [29,38].

### Co-endemicity

Polyparasitism is believed to prime the body increasing its susceptibility to coinfection with other parasites, consequently playing a vital role in the development of morbidity [14,38,51]. Our findings showing lower egg burden *in S. haematobium*-*S. mansoni* coinfection differed with other studies showing higher egg burden due to coinfection [52]. Moreover, infection with *S. haematobium* was not associated with *S. mansoni* infection. Such inconsistencies have also been previously reported especially in large scale studies [53,54]. Differences in local transmission of both *S. mansoni* and *S. haematobium* mono and coinfections between large and small focal surveys has been suggested to be the main reason for this disparity [52]*.*

The prevalence of *S. haematobium* mono infections was notably lower than *S. mansoni* in all the districts, with infections majorly being mild and some severe cases in Migori district. However, if left untreated, the mild to severe infections could lead to adverse pathological effects in the later decades of life. The long term detrimental effect is characterized by ureter and bladder outflow obstruction, kidney malfunction or a predisposition of developing squamous cell carcinoma of the bladder [55,56]. In women, *S. haematobium* ova can also be transmitted to the genital organs [57], causing pathological lesions that are exacerbated by higher infection intensities [57-62]. Female genital schistosomiasis is currently attracting increased attention after being associated with increasing the susceptibility to infection with HIV and even persistent strains of Human papillomavirus (HPV), an etiologic agent for cervical cancer [63-66]. Infections with the persistent HPV strains could lead to cervical neoplasia even after *S. haematobium* has been successfully controlled [66]. Therefore, with the recorded high prevalence of *S. haematobium* infections in our study, it is important to control both schistosome infection and its associated spillover effects, including FGS.

This information has an urgent benefit in addressing concerns related to FGS amongst girls and women, given that national deworming programmes mainly target school going children, leaving the once infected women at risk of further complications.

### Implications for mass drug administration programs

Just after this study was completed in early June 2013, the Kenya National deworming programme started conducting mass drug administration with praziquantel, in addition to the annual school based albendazole deworming programme that was launched in 2009. Information provided in this survey will therefore be an important tool in identifying particular high prevalence areas (with more than 10% and 20% for schistosomiasis and STHs, respectively) where impact of MDA need to be closely monitored. It can also serve as a basis for further investigation on the best MDA approach that could be implemented for control of schistosomiasis.

## Conclusions

In summary, our findings identify a number of inland transmission sites for schistosomiasis in south Nyanza, western Kenya, mainly found near particular water bodies (ponds, water-points, streams, dams or rivers) in Rachuonyo, Migori and Homabay districts. This has important implications in planning and implementing control interventions in these regions, and can be utilized in the recently launched 5 year National Strategic Plan against NTDs, especially in extending interventions to adult populations.

## Abbreviations

WHO: World Health Organization; GIS: Geographic information systems; GPS: Global positioning system; STHs: Soil-transmitted helminths; MDA: Mass drug administration; DVBNTD: Division of vector-borne and neglected tropical diseases.

## Competing interests

The authors declare that they have no competing interests.

## Authors’ contributions

The study was designed by HCS, MRO and PNMM. HCS and GM coordinated the field and lab activities. MO and HCS participated in analyzing the GIS data and generating maps. HCS compiled and analyzed all the other data. Scientific guidance in data collection, planning and carrying out the daily field and lab work was provided by MRO and PNMM. The manuscript was prepared by HCS, and the final version was read and approved by all the authors.
